# Multineuronal spike sequences repeat with millisecond precision

**DOI:** 10.3389/fncir.2013.00112

**Published:** 2013-06-21

**Authors:** Koki Matsumoto, Tomoe Ishikawa, Norio Matsuki, Yuji Ikegaya

**Affiliations:** ^1^Graduate School of Pharmaceutical Sciences, The University of TokyoTokyo, Japan; ^2^Center for Information and Neural NetworksSuita City, Osaka, Japan

**Keywords:** spontaneous activity, calcium imaging, action potentials, spike sequences, hippocampus, ripple

## Abstract

Cortical microcircuits are nonrandomly wired by neurons. As a natural consequence, spikes emitted by microcircuits are also nonrandomly patterned in time and space. One of the prominent spike organizations is a repetition of fixed patterns of spike series across multiple neurons. However, several questions remain unsolved, including how precisely spike sequences repeat, how the sequences are spatially organized, how many neurons participate in sequences, and how different sequences are functionally linked. To address these questions, we monitored spontaneous spikes of hippocampal CA3 neurons *ex vivo* using a high-speed functional multineuron calcium imaging (fMCI) technique that allowed us to monitor spikes with millisecond resolution and to record the location of spiking and non-spiking neurons. Multineuronal spike sequences (MSSs) were overrepresented in spontaneous activity compared to the statistical chance level. Approximately 75% of neurons participated in at least one sequence during our observation period. The participants were sparsely dispersed and did not show specific spatial organization. The number of sequences relative to the chance level decreased when larger time frames were used to detect sequences. Thus, sequences were precise at the millisecond level. Sequences often shared common spikes with other sequences; parts of sequences were subsequently relayed by following sequences, generating complex chains of multiple sequences.

## Introduction

The brain uses a limited number of neurons to process virtually unlimited patterns of information from external environments. Therefore, individual neurons are unlikely to independently process specific information, and it is more plausible that they cooperatively form subgroups that work as functional units. This idea, called the “cell assembly” hypothesis (Hebb, 1949; Harris, 2005; Buzsaki, 2010), leads to two important predictions about neuronal circuit operation. First, a given neuron can participate in two or more cell assemblies. Second, the synapse weight between two given neurons is modifiable over time. More specifically, the weight is strengthened when the neurons work cooperatively, otherwise it is weakened. This bidirectional synaptic plasticity has been experimentally proven by studies showing that cortical synapses are capable of exhibiting long-term potentiation (LTP) and long-term depression (LTD) of synaptic transmission. These two features suggest that neuronal networks self-organize through reorganization of neuronal connectivity due to ongoing external stimuli and are thereby functionally compartmentalized to form cell assemblies.

Synchronous activity among multiple neurons is regarded as one of the simplest aspects of cell assembly dynamics, not only because it triggers the induction of synaptic plasticity but also because it is realized through synchronization between groups of presynaptic neurons (Takahashi et al., 2010). At a more microscopic level, synchronized activity is often composed of sequential activation of multiple neurons. Indeed, such multineuronal spike sequences (MSSs) are known to exist during sensory-evoked and spontaneous network activity at frequencies greater than chance (Abeles and Gerstein, 1988; Prut et al., 1998; Abeles and Gat, 2001; Ikegaya et al., 2004; Luczak et al., 2007). MSSs are conceptually consistent with the so-called “synfire chains” hypothesis, in which neurons form several layers through which synchronized spikes can stably propagate with temporal precision, therefore generating a chain of spikes across neurons (Abeles, 1991). Although MSSs have been reported in different brain preparations, including the neocortex of monkeys and rodents *in vivo* and *in vitro*, some reports still cast doubt on the existence of MSSs (Oram et al., 1999; Baker and Lemon, 2000; Mokeichev et al., 2007).

We focus the present work on the hippocampus. One of the reasons for this selection is that the hippocampus spontaneously emits sharp waves-ripples (SW-Rs), a transient form of high-frequency field oscillations. SW-Rs primarily occur during slow-wave sleep and quiet awake states (Buzsaki et al., 1983). During SW-Rs, neurons that were previously involved in behavioral exploration, called place cells, increase their firing rates sequentially in the same or opposite order of which those neurons were activated during the behavioral exploration (Lee and Wilson, 2002; Harris et al., 2003; Foster and Wilson, 2006; O'Neill et al., 2006; Diba and Buzsaki, 2007; Pastalkova et al., 2008; Davidson et al., 2009). Therefore, the hippocampus may be a good model to study MSSs. *In vitro* slices prepared from the hippocampus are also reported to emit spontaneous SW-Rs (Norimoto et al., 2012; Sun et al., 2012), and the slices may produce MSSs. If it were the case, it would be easy to analyze the properties of MSSs because the *in vitro* experimental system is more accessible and manipulatable than the *in vivo* system.

Functional multineuron calcium imaging (fMCI) is an optical technique that monitors spikes of neurons *in situ* through spike-evoked somatic calcium transients. We have optimized the method for hippocampal slice cultures (Takahashi et al., 2007, 2011). In the CA3 region of organotypically cultured slices, the probability of a synaptic connection between randomly selected adjacent pyramidal cells is approximately 25%. This ratio is higher than that reported in acute slice preparations (2–8%) (Miles and Wong, 1983; Smith et al., 1995). In acute hippocampal slices, however, 75–90% of the axons of CA3 pyramidal neurons are cut, even in slices as thick as 500 μm (Gomez-Di Cesare et al., 1997). In contrast, cultured networks self-restore their complexity to a realistic extent. Indeed, levels of spontaneous excitatory or inhibitory postsynaptic currents are similar between *ex vivo* and *in vivo* hippocampal neurons (Takahashi et al., 2010). Moreover, we have demonstrated, by using an optical synapse mapping technique, that in such restored CA3 networks, pyramidal cells are nonrandomly connected to generate diverse repertoires of synchronized activity, like the *in vivo* conditions (Takahashi et al., 2010).

## Materials and methods

### Animal

Experiments were performed with the approval of the animal experiment ethics committee at the University of Tokyo (approval number: 19-43, P21-6) according to the University of Tokyo guidelines for the care and use of laboratory animals.

### Slice culture preparations

Entorhino-hippocampal organotypic slices were prepared from 7-day-old Wistar/ST rats (SLC, Shizuoka, Japan) as previously described (Koyama et al., 2007). Briefly, rat pups were anesthetized by hypothermia and decapitated. The brains were removed and placed in aerated, ice-cold Gey's balanced salt solution supplemented with 25 mM glucose. Horizontal entorhino-hippocampal slices were made at a thickness of 300 μm using a vibratome (DTK-1500, Dosaka, Kyoto, Japan). The slices were placed on Omnipore membrane filters (JHWP02500, Millipore, Bedford, MA, USA) and incubated in 5% CO_2_ at 37°C. The culture medium, which was composed of 50% minimal essential medium (Invitrogen, Gaithersburg, MD, USA), 25% Hanks' balanced salt solution, 25% horse serum (Cell Culture Laboratory, Cleveland, OH, USA), and antibiotics, was changed every 3.5 days. Experiments were performed on days 7–11 *in vitro*.

Although slice cultures are known to form abnormal connections that very rarely exist under normal conditions, such as CA1-to-CA1, CA1-to-CA3, and CA3-to-dentate gyrus connections (Gahwiler et al., 1997; De Simoni et al., 2003), there are few of these aberrant connections in our slice culture preparations. Investigation using reverse optical trawling, a synapse mapping technique (Sasaki et al., 2009), demonstrated that these abnormal connections are less than 0.5% of the total connections and that an overwhelming number of connections project to their normal targets. This result is most likely because the entorhinal cortex was not dissected out in our preparations. Lesions of the entorhinal cortex are known to result in abnormal sprouting and reorganization of hippocampal networks *in vivo* and *ex vivo* (Laurberg and Zimmer, 1981; West and Dewey, 1984).

### Functional multineuron calcium imaging

**Figure 1:**
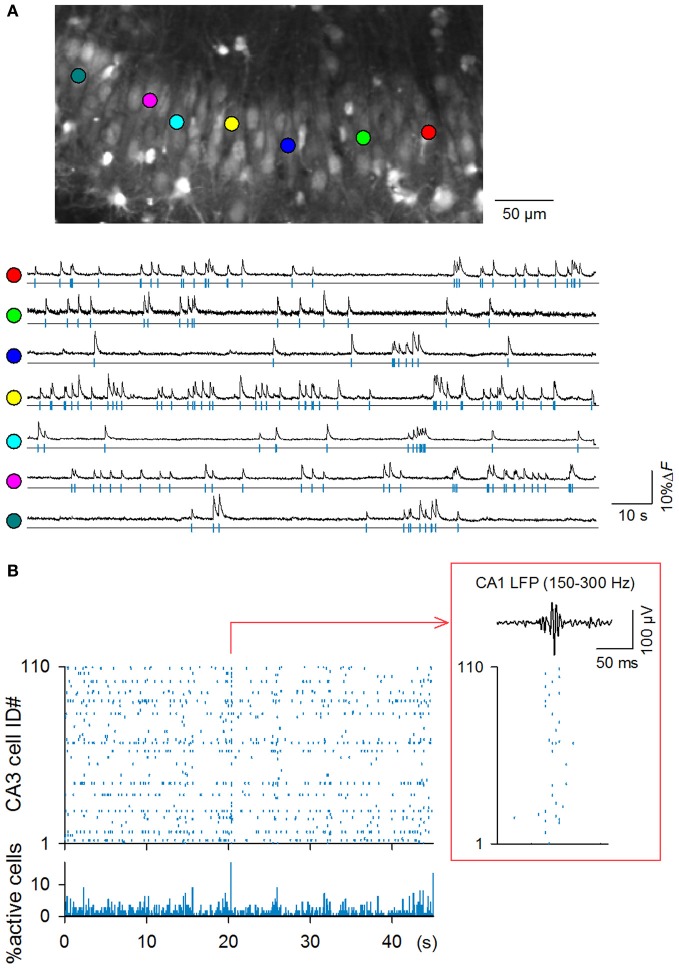
**High-speed fMCI of spontaneous CA3 network activity. (A)** *Top*: representative confocal image of the CA3 stratum pyramidale in an OGB1-loaded hippocampal slice culture. *Bottom*: Time changes in the OGB1 fluorescence intensity in the somata of 7 randomly selected neurons indicated by the colored circles in the top photograph. **(B)** Representative rastergram of all 110 CA3 neurons (top) and time histogram of the percentage of active neurons (bin = 10 ms). A large synchronization is time-expanded in the inset and is displayed together with 150–300 Hz band-passed LFPs simultaneously recorded from the CA1 pyramidal stratum. Synchronization was accompanied by SW-R-like oscillations.

Slices were incubated with 2 ml of dye solution at 37°C for 1 h (Takahashi et al., 2011). The dye solution was aCSF containing 0.0005% Oregon Green 488 BAPTA-1AM (OGB-1AM), 0.01% Pluronic F-127, and 0.005% Cremophor EL. After a 1-h recovery, a slice was transferred to a recording chamber. Images were acquired at 500 frames/s with a Nipkow-disk confocal unit (CSUX-1, Yokogawa Electric, Tokyo, Japan), a back-illuminated electron-multiplying charge-coupled device (EM-CCD) camera (iXon DV860, Andor, Belfast, Northern Ireland, UK), a water-immersion objective lens (16×, 0.80 NA, Nikon, Tokyo, Japan), and Solis software (Andor). Fluorophores were excited at 488 nm with an argon laser (10–15 mW, 532-BS-AO4, Omnichrome, Chino, CA, USA) and visualized with a 507-nm long-pass emission filter. In each cell body, the fluorescence change ΔF/F was calculated as (*F*_*t*_–*F*_0_)/*F*_0_, where *F*_*t*_ is the fluorescence intensity at frame time *t*, and *F*_0_ is baseline (Figure 1A). Spike timing was defined as the onset of individual calcium transients with an automatic machine-learning algorithm that can accurately detect spike timing within one frame jitter (Sasaki et al., 2008). In some experiments, picrotoxin was bath-applied to prevent fast inhibitory synaptic transmission. Picrotoxin (purchased from Sigma-Aldrich, St. Louis, MO) was dissolved in aCSF at the final concentration of 50 μM and perfused to slices. Imaging was started 30 min after the perfusion onset.

### Local field potential recordings and ripple detection

In a single experiment, CA1 local field potentials (LFP) were recorded during fMCI monitoring of the calcium activity of CA3 neurons. Glass pipettes were filled with 2 M NaCl and placed in CA1 stratum pyramidale. To extract the SW-R activity, the recorded data were band-pass filtered at 150–300 Hz. SW-R-like events were automatically detected based on their oscillatory powers and durations; the root mean square (3-ms window) of the band-passed signal was used to detect SW-Rs of 10 ms duration with a power threshold of 5 standard deviations (SDs).

### MSS detection

We used a template-matching algorithm to search for MSSs (Ikegaya et al., 2004). We first selected cells that showed more than one calcium transient. After determining the reference calcium levels of a reference cell (*cell*_1_), we designated a vector consisting of a set of cells and relative timings of their calcium events as follows: (*cell*_2_, …, *cell*_*N*_, *t*_2_, *t*_3_,…, *t*_*N*_), where *t*_*i*_ denotes the delay of the event in *cell*_*i*_ after the reference event. *t*_*i*_ was limited to less than 500 ms. This vector was used as a template and was slid along the successive events of *cell*_1_ throughout the recording session. If more than two elements were identical between any template pairs, we regarded the matched elements as an MSS. Each mismatched spike configuration was used as another template in a subsequent scan. Thus, every event was considered part of a template MSS, and each template occurred at least once. Unless otherwise specified, one frame jitter (2 ms) was allowed so that the total number of MSSs satisfied statistical demands (Kendall et al., 1994).

### Surrogate data

To determine whether MSSs or their structures can arise from a stochastic process, we created surrogate raster plots using a Monte Carlo resampling method (Ikegaya et al., 2004). Each calcium event was exchanged between a pair of cells, maintaining their relative timings (Figure 3A). This procedure was repeated for all calcium transients in each raster plot. This randomization preserves the event frequencies of individual cells and the population modulation of event timings, such as network synchronization. In each shuffled surrogate, we searched MSSs using the same detection algorithm. Twenty surrogates were generated for each dataset, and the averages across the 20 surrogates were defined as the chance level.

### Asynchrony index

The temporal sparseness of network activity during the observation period was captured by a normalized Shannon index, termed asynchrony index (Usami et al., 2008; Mizunuma et al., 2009; Ujita et al., 2011). The Shannon index quantifies the dispersion of components in a histogram and is generally defined as −∑_*i*_ (*k*_*i*_/K) *log*_2_ (k_*i*_/K), where *K* is the total number of components, and *k*_*i*_ is the number of components in the *i*-th bin. This definition of diversity is conceptually equivalent to Shannon's entropy. Because this index is sensitive to *K* and the bin size, SI has often been normalized with the maximal value and other standard values to compare groups. Here we normalized Shannon index with the maximal (*SI*max) and minimal values (*SI*min) that can be taken in the distribution of the same number of spikes in the raster plot. *SI*max and *SI*min were obtained through data shuffle with maintaining *K* and bin; *SI*max is given when components are as evenly redistributed over the time axis as possible, whereas *SI*min is given when components are as temporally biased as possible. Then normalized Shannon index is defined as *(SI–SI*min)/(*SI*max–*SI*min). Thus, it takes a value from 0 to 1, with higher values being more dispersive.

### Data representation

We reported all averaged values as the mean ± SDs.

## Results

### High-speed imaging of spiking CA3 networks *ex vivo*

Hippocampal slice cultures were incubated in OGB1AM, and OGB1-loaded neurons were imaged from the CA3 stratum pyramidale at 500 Hz using a spinning-disk confocal microscope and a high-speed EM-CCD camera (Takahashi et al., 2011). The microscopic field covered an area of approximately 350 × 200 μm (Figure 1A) and contained an average of 91.7 ± 26.7 neurons (mean ± SD of 9 videos, ranging from 60 to 137 neurons). Each video was 130 s in length, and a total of 9 videos were recorded from 9 slices (*n* = 9 rat pups borned from 9 mothers).

In all 9 videos, spontaneous activity was evident; among a total of 825 neurons in 9 videos, 757 neurons (91.8%) exhibited at least one spike during the observation period. The mean firing rates of active neurons were 0.25 ± 0.29 Hz (*n* = 9 videos), ranging from 0.008 to 2.33 Hz. Therefore, spontaneous activity was sparse as a whole. Nevertheless, neurons occasionally exhibited synchronization at the population level (Figure 1B); synchronization that recruited more than 5% or 10% of the total neurons occurred at frequencies of 2.46 ± 3.0 or 0.26 ± 0.52 per min, respectively (*n* = 9 videos, bin = 2 ms = one frame).

We succeeded in simultaneous LFP recording in one single video; note that in general, LFP recording is technically difficult in slice cultures, most likely because neurite reorganization during cultivation slightly alters the fine layer structure of the hippocampus, collapsing the net dipole moment generated by synaptic activity or because cultured networks may be heterogenous from preparation to preparation (e.g., see Figure 4). In the single dataset, we found that synchronous activity was always accompanied by SW-R-like high-frequency oscillations (Figure 1B, inset).

### MSSs with millisecond precision

**Figure 2:**
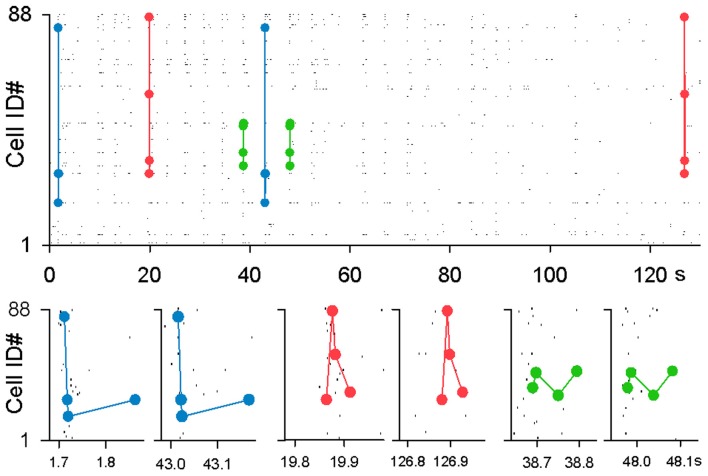
**Examples of MSSs**. Three representative MSSs are merged in a single rastergram and magnified in time in the bottom panels.

**Figure 3:**
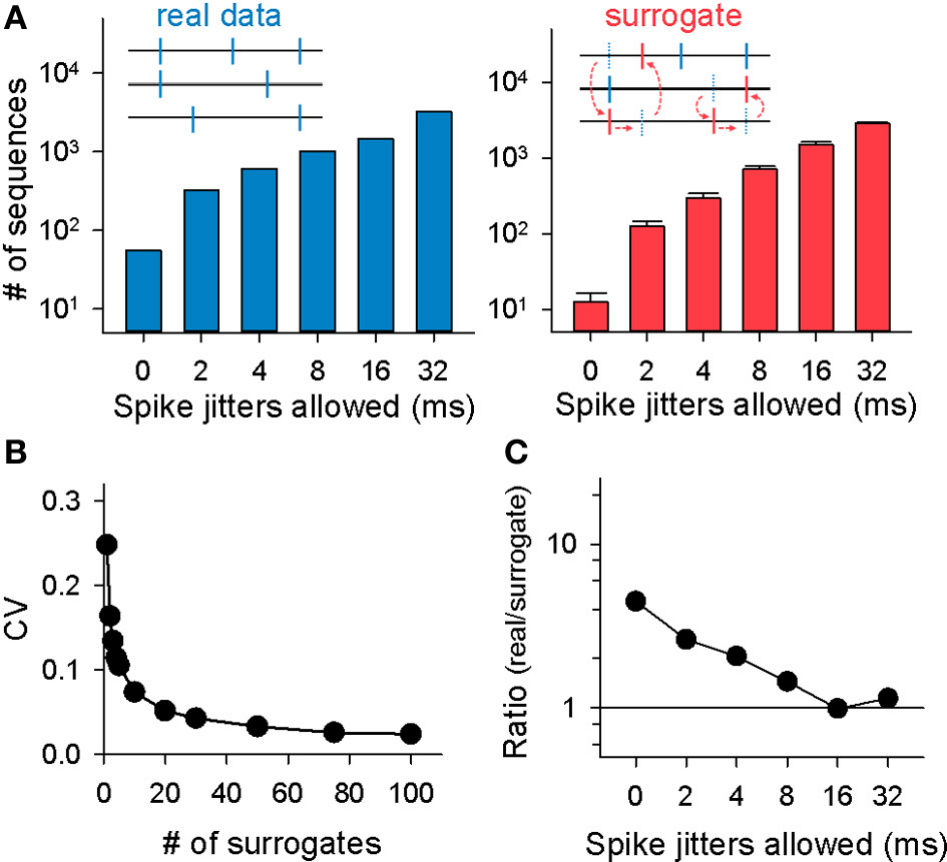
**Monte Carlo evaluation for the significance of MSSs. (A)** *Left*: the total numbers of MSSs in the rastergram shown in **(A)** are plotted as a function of spike-time jitters allowed for MSS detection. *Right*: MSSs were identified in the same way in spike-shuffled rastergrams. Data are the mean ± SDs of 20 surrogates. **(B)** Relationship between the CVs of the number of MSSs detected in surrogates and the number of surrogates generated. **(C)** Ratios of the numbers of MSSs in the original (real) rastergram to the mean numbers of MSSs in 20 randomized surrogates. Data are the same as **(B)**.

We detected MSSs. The maximal length of an MSS, i.e., the time interval between the first spike and the last spike, was set to be 500 ms. Three examples of MSSs found in a raster plot are shown in Figure 2. Not surprisingly, the number of MSSs depended on the spike time jitters allowed to detect MSSs (Figure 3A left). MSSs increased in number when jitters were increased from 0 ms (0 frame) to 32 ms (16 frames); note that 0-ms jitter (= 0 frames) had a time window of 2 ms due to 500-Hz imaging.

**Figure 4:**
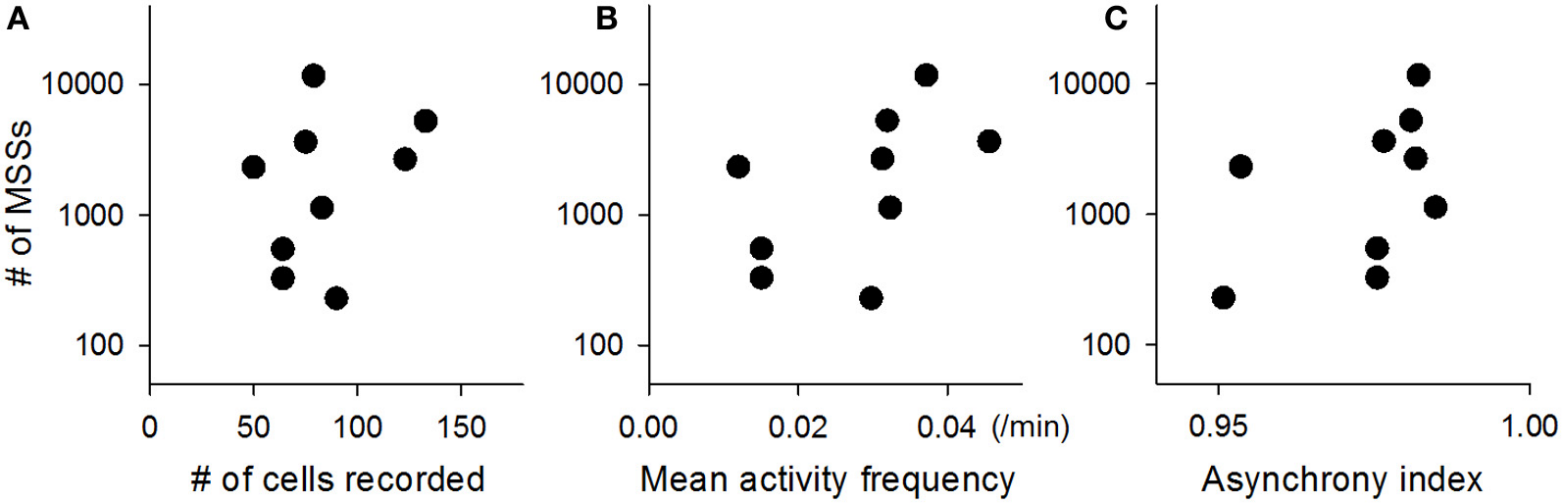
**Across-video variations of the number of MSSs**. The total number of MSSs varied among datasets and thus is plotted against the total number of neurons recorded in the corresponding videos **(A)**, the mean frequency of activity of those neurons **(B)**, and the synchrony index of the corresponding videos **(C)**. Each dot indicates a single video. *n* = 9 videos.

The total number of MSSs varied among videos, probably because the network states fluctuate over time and are not identical among preparations (Sasaki et al., 2007). The total number of MSSs in each video (2-ms jitter allowed) was plotted against the total number of neurons recorded in the corresponding videos (Figure 4A), the mean activity frequency of those neurons (Figure 4B), and the asynchrony index of the corresponding videos (Figure 4C); note that the asynchrony index is a normalized Shannon index for spike dispersion along the time axis (Usami et al., 2008; Mizunuma et al., 2009; Ujita et al., 2011). Thus, we investigated the statistical significance of MSSs by comparing the number of MSSs to its stochastic level. To estimate the stochastic level, we created surrogate raster plots by randomly exchanging spikes across neurons. Specifically, a single spike of a randomly selected neuron was swapped with a spike of another randomly selected neuron without changing their absolute spike timings, and this swapping procedure was repeated until all spikes in the original dataset were exchanged (Figure 3A right, inset). This shuffling method preserved both the firing rates of individual neurons and the level and frequency of network synchronization. Therefore, comparing an original dataset with these surrogates makes it possible to examine whether MSSs are actively generated by complex network dynamics or are merely a mathematically natural consequence of the firing rates of individual neurons.

**Figure 5:**
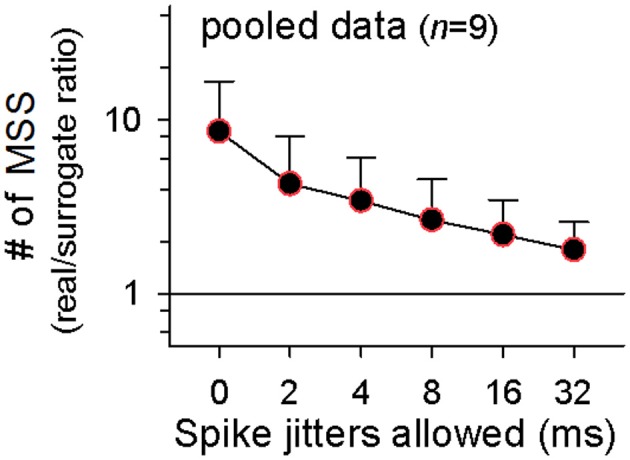
**Millisecond precision of MSSs**. Summary of 9 videos. Color-edged circles are 5% significant data points between real datasets and surrogates (paired *t*-test). The data are the mean ± SDs.

The coefficient of variation (CV) for the number of MSSs detected in surrogates depended on the number of surrogates generated. The CV rapidly decreased as a function of the number of surrogates; it dropped to about 0.05 by 20 surrogates, and thereafter, it was kept almost constant (Figure 3B, 2-ms jitter allowed). To reduce computation burden with preserving statistical stability, therefore, we searched MSSs in 20 surrogates generated from each raster plot. MSSs in the surrogates also increased in number with spike jitter timing (Figure 3A right). We thus plotted the ratio of the MSS number in the real dataset to that in the surrogates as a function of jitters. The ratio peaked at a jitter of 0 ms and decreased gradually with increasing jitters (Figure 3C). We repeated this procedure for all other datasets and found similar results in the pooled data (*n* = 9 videos; Figure 5), that is, the mean real-to-surrogate ratio is a simple reduction function of spike jitters. Based on these results, we reached two fundamental conclusions: (1) MSSs emerge more frequently than expected by chance and therefore cannot be explained by a stochastic process, and (2) relative spike times within MSSs are maintained at the millisecond level.

### Internal structures of MSSs

In the following analyses, we used MSSs detected at a spike jitter of 2 ms. Although the real-to-surrogate ratios of MSSs was maximal at a jitter of 0 ms, the absolute number of MSSs detected at 0 ms was substantially smaller than at 2 ms. To enhance statistical accuracy, we selected the 2-ms jitter in this study. Note that the MSS properties described below were fundamentally consistent and exhibited similar tendencies at jitters of 0, 2, 4, and 8 ms (data not shown).

**Figure 6:**
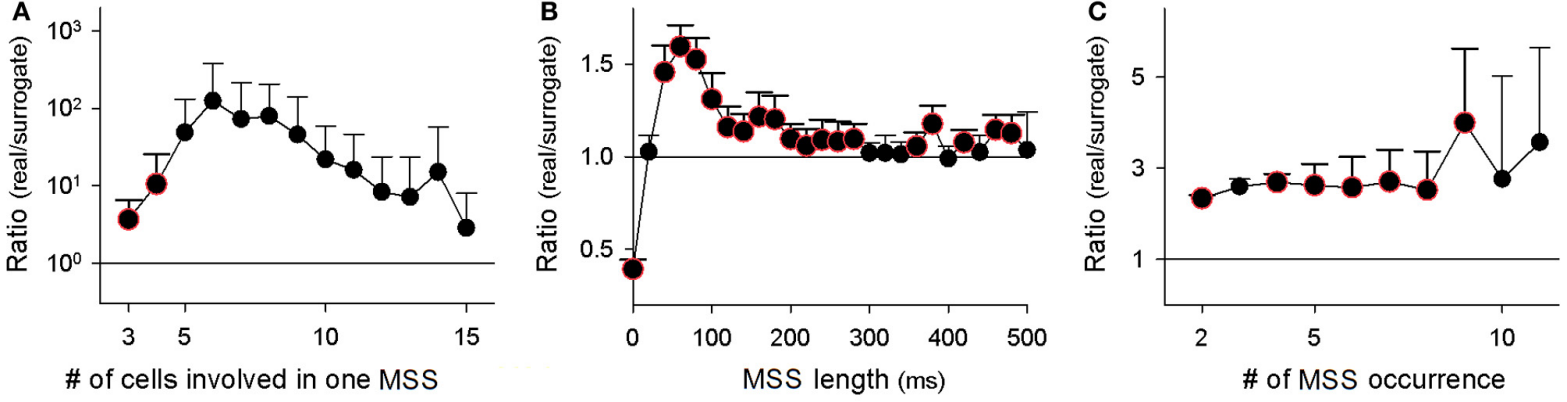
**Characterization of MSSs. (A–C)** The number of cells that participated in individual MSSs **(A)**, the duration that individual MSS events persisted (i.e., the time intervals between the first and last spikes within MSSs) **(B)**, and the number of repetitions of MSSs **(C)** are shown as ratios of values in real datasets to the mean value of 20 randomized surrogates. Color-edged circles are 5% significant data points between real datasets and surrogates (paired *t*-test). The data are the mean ± SDs of 9 videos.

At a jitter of 2 ms, we identified a total of 27,804 MSSs in 9 videos. Among the 825 total neurons, 624 neurons (75.6%) participated in at least one MSS. On average, single MSSs contained 3.4 ± 1.1 neurons, persisted for 107.9 ± 158.6 ms (i.e., the MSS length, defined as the time interval between the first and last spike), and were repeated 2.1 ± 1.1 times during the observation periods. We compared these values to those found in surrogate datasets. The real-to-surrogate ratio of the number of neurons involved in single MSSs was consistently higher than 1 and peaked at 5–8 neurons (Figure 6A). As to the MSS length, the real-to-surrogate ratios of the number of MSSs were significantly higher than 1, and roughly in the range of 40–280 ms, peaking at 60 ms (Figure 6B). The real-to-surrogate ratios of the number of MSSs were independent of MSS repeat numbers and were consistently higher than 1 (Figure 6C).

**Figure 7:**
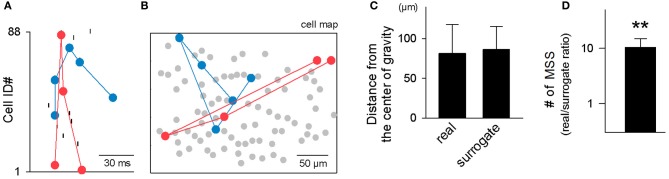
**Spatial dispersion of MSSs. (A,B)** Two representative MSSs in a rastergram **(A)** and in a cell map **(B)**. **(C)** The mean Euclidean distance from individual cells that participated in a MSS to the center of gravity of all cells that participated in the MSS was compared surrogates in which the same number of cells in the original MSS were selected randomly from the same cell map. This comparison was repeated for 27,804 MSSs in 9 videos, and data are shown as the mean ± SDs. **(D)** The number of MSSs was still overrepresented in surrogates generated by activity shuffling in which spatial configurations were preserved. ^**^P < 0.01, paired *t*-test. The data are the mean ± SDs of 9 videos.

Two examples of MSSs (Figure 7A) are shown in a cell map (Figure 7B). We examined whether the neurons involved in each MSS were spatially clustered. For each MSS, we computed the center of gravity for the locations of all neurons that participated in the MSS and calculated the mean distance from these neurons to the center of gravity. If the MSS is spatially clustered, the mean distance will be smaller than chance. The chance values were estimated by 100 surrogates of pseudo-MSSs generated by the same number of neurons randomly selected from the original map. The mean distance from the center of gravity did not differ between real and surrogate MSSs [*t*_(27, 804)_ = 1.08, *P* = 0.28, paired *t*-test; Figure 7C]. Thus, MSS-participating neurons are spatially dispersed at the stochastic level.

**Figure 8:**
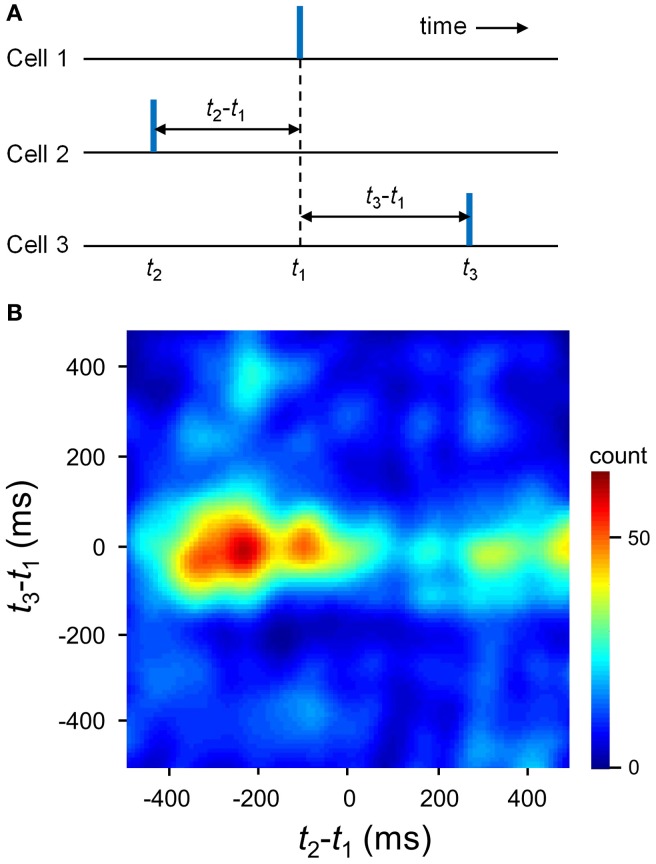
**Pairwise correlogram of precisely repeating triplets. (A)** For every trio of neurons, a spike triplet is described by two inter-spike intervals (*t*_2_–*t*_1_ and *t*_3_–*t*_1_). **(B)** Count matrix for a representative triplet of neurons, indicating the probability of different inter-spike interval combinations.

Here we returned to the statistical issues about MSS existence. Our spike shuffling method did not consider the spatial organization of cells that participated in MSSs. We thus created surrogates using another shuffling method; for each MSS, spikes were preserved for only one MSS appearance, while the other spikes constituting the MSS were randomly exchanged within the MSS. This procedure did not collapse the spatial organization of cells that exhibited MSSs. Using this shuffling method, we again confirmed that MSSs were overrepresented relative to chance (Figure 7D, *n* = 9 videos). However, because these Monte Carlo-shuffling evaluations did not completely abolish statistically innate problems, such as false-negative errors and false-positive errors, we also adopted a completely different approach to validate MSSs without spike shuffling; MSSs are embossed in pairwise correlograms among spike triplets occurring across three distinct cells (Luczak et al., 2007). For each cell trio, one cell was designated the trigger for calculation of the joint distribution of spike times of the other two (Figure 8A). A clear dense peak was observed at *t*_2_–*t*_1_ = −231 ms and *t*_3_–*t*_1_ = −12 ms in this distribution (*n* = 180 triplet spikes), suggesting that a particular sequence occurred preferentially (Figure 8B).

**Figure 9:**
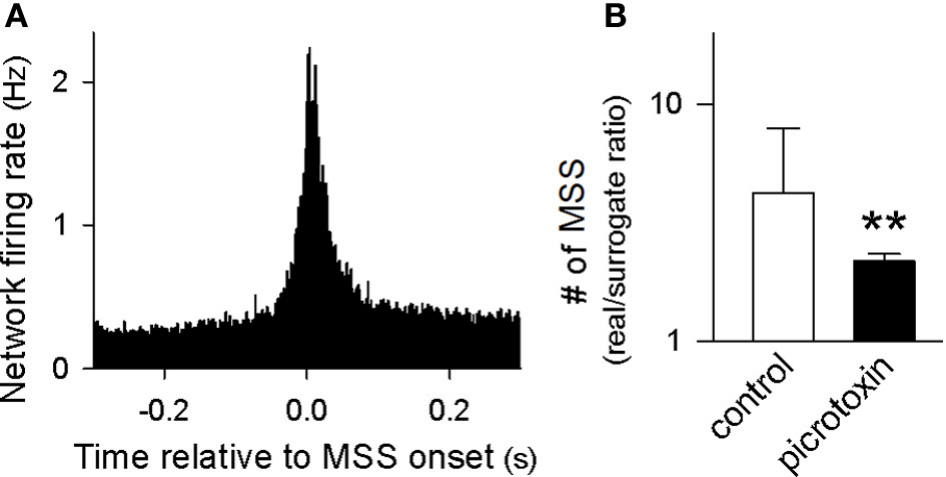
**Mechanisms of MSSs. (A)** Peri-MSS time histogram of the mean firing rates of all neurons monitored in 9 videos. The firing rates were aligned at the time relative to the onset of 27,804 individual MSSs. **(B)** The number of MSSs relative to chance were decreased by bath application of 50 μM picrotoxin. ^**^P < 0.01 versus control, Student's *t*-test. The data are the mean ± SDs of 9 videos.

We sought to examine the timing of MSS appearance relative to the entire network activity. Figure 9A demonstrates the peri-MSS time histogram of the mean firing rates of all neurons in the video. In the peri-MSS time histogram, we aligned the first spikes in individual MSSs at time 0. Data were pooled from 27,804 MSSs in 9 videos. The histogram revealed that the neuronal network transiently increased the global firing rate during MSSs. This transient synchronization persisted for approximately 100 ms, which corresponded to the durations of SW-Rs (Buzsaki et al., 1983).

During SW-Rs, excitatory neurons and inhibitory neurons were both activated at particular phases in a cell type-specific manner (Klausberger et al., 2003). To examine the involvement of GABAergic transmission, we perfused slices with 50 μM picrotoxin, a GABA_A_ receptor channel inhibitor. Bath application of picrotoxin reduced MSSs, as compared to control solution (Figure 9B). Thus, MSSs do not represent simple spike chains via excitatory synapses, but rather, they are more likely to emerge actively from network-coordinated excitatory and inhibitory balance.

### Biased MSS participation of individual neurons

**Figure 10:**
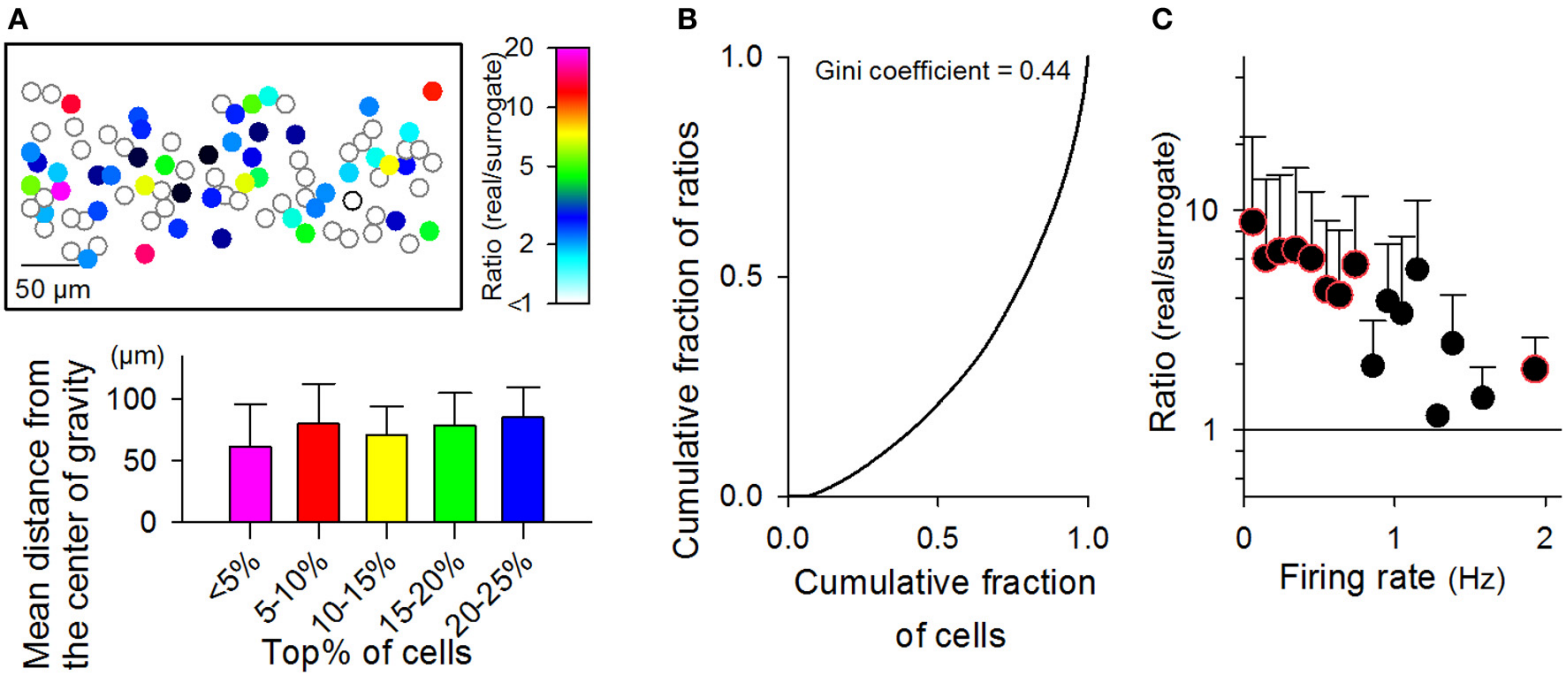
**Relationship between the firing rates of neurons and their MSS participations. (A)** *Top:* a cell map representing the number of individual cells participating in MSSs during the observation period. Data are shown as the ratio of the real rastergrams to their randomized controls. *Bottom:* The mean Euclidean distance to the center of gravity from individual cells with the highest *x*% of the real-to-surrogate ratio. The ranges of *x* values are indicated in the abscissa. For example, in the case of 5–10%, we selected cells with the real-to-surrogate ratios that ranged from the top 5 to the top 10%, calculated the center of gravity of those selected cells, and measured the mean distance from those cells to their center of gravity. **(B)** A Lorenz curve representing the proportion of real-to-surrogate ratios assumed by the proportion of cells with the lowest ratios. A Lorenz curve is used to show the degree of “inequality” of a distribution defined by two variables (herein, the real-to-surrogate ratio of individual cells *versus* the number of cells with a given real-to-surrogate ratio). The inequality can be represented by a Gini coefficient, a number between 0 and 1, where perfect equality has a Gini coefficient of zero, and absolute inequality yields a Gini coefficient of 1. In this case, the Gini coefficient was 0.44. **(C)** The ratios of the numbers of MSS participations of individual cells in real datasets to the mean values in 20 randomized surrogates are plotted against their mean firing rates. Color-edged circles are 5% significant data points between real datasets and surrogates (paired-*t* test). The data are the mean ± SDs of 9 videos.

The same neurons were often recruited in different MSSs. On average, individual cells participated in 81.8 ± 282.2 MSSs; however, the frequency of MSS participation varied among neurons. The representative cell map shown in Figure 10A represents the real-to-surrogate ratios of the number of MSS participations with a pseudo-colored scale, indicating that some neurons frequently participated in MSSs, whereas others did not. In Figure 10B, we plotted the Lorenz curve of these frequency ratios (*n* = 825 neurons in 9 videos). The Gini coefficient was 0.44, indicating that neurons are not homogeneous in terms of MSS participation.

To investigate the spatial organization of MSS-participating neurons, we selected neurons that scored in the top 5% of the frequency ratios and computed the mean distance from these cells to their center of gravity (Figure 10A bottom). We also calculated the mean distances for the top 5–10, 10–15, 15–20, and 20–25% of the neurons and found that they did not differ among these groups [*P* = 0.43, *F*_(4, 40)_ = 0.97, One-Way ANOVA, Figure 10A bottom]. Thus, the spatial distribution of neurons is unlikely to depend on the frequency of MSS participation.

We plotted the frequency of MSS participation against the firing rates of the corresponding neurons (Figure 10C). The real-to-surrogate ratios of the MSS participation frequency decreased with the firing rates. These results indicate that frequently firing neurons do not necessarily participate frequently in MSSs, whereas rarely firing neurons seem to contribute more significantly to MSSs.

**Figure 11:**
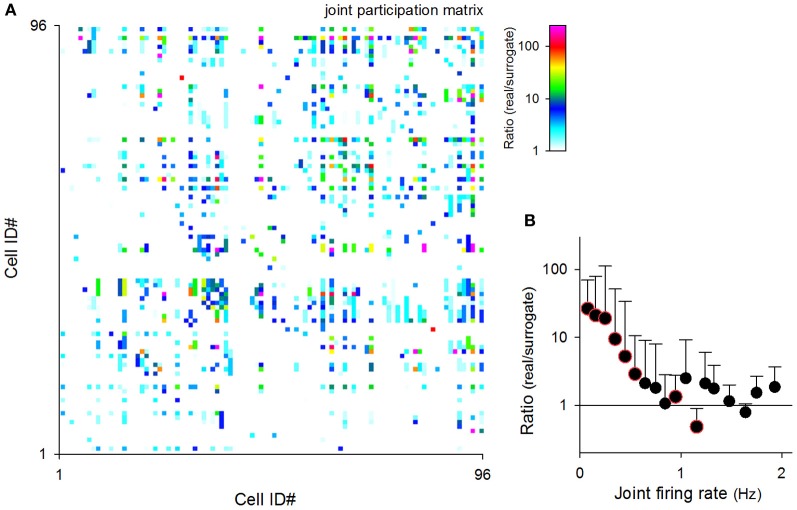
**Relationship between the joint firing rates of neuron pairs and their MSS joint participations. (A)** A pseudo-colored matrix representing the numbers of joint participations by two given cells. Data are shown as the ratio of the real rastergrams to their randomized controls. **(B)** The ratios of the number of MSS co-participations of cell pairs in real datasets to the mean value of 20 randomized surrogates were plotted against their mean joint firing rates. Color-edged circles are 5% significant data points between real datasets and surrogates (paired-*t* test). The data are the mean ± SDs of 9 videos.

We then conducted a similar analysis for neuron pairs. Joint participation was defined as when two given neurons simultaneously participated in the same MSS. We counted the number of joint participations for all possible pairs of neurons in each video. Figure 11A shows a representative matrix of the real-to-surrogate ratios of the frequency of joint participation of 96 neurons in a single video. Like the behaviors by single neurons, some neuron pairs co-participated frequently in MSSs, whereas other pairs did not. Moreover, the real-to-surrogate ratios of the joint participation frequency decreased with the joint firing rates of the neuron pairs (Figure 11B); note that the joint firing rates were defined as (*f*_*i*_ × *f*_*j*_)^1/2^, where *f*_*i*_ and *f*_*j*_ are the firing rates of cell_*i*_ and cell_*j*_.

### MSS chains

**Figure 12:**
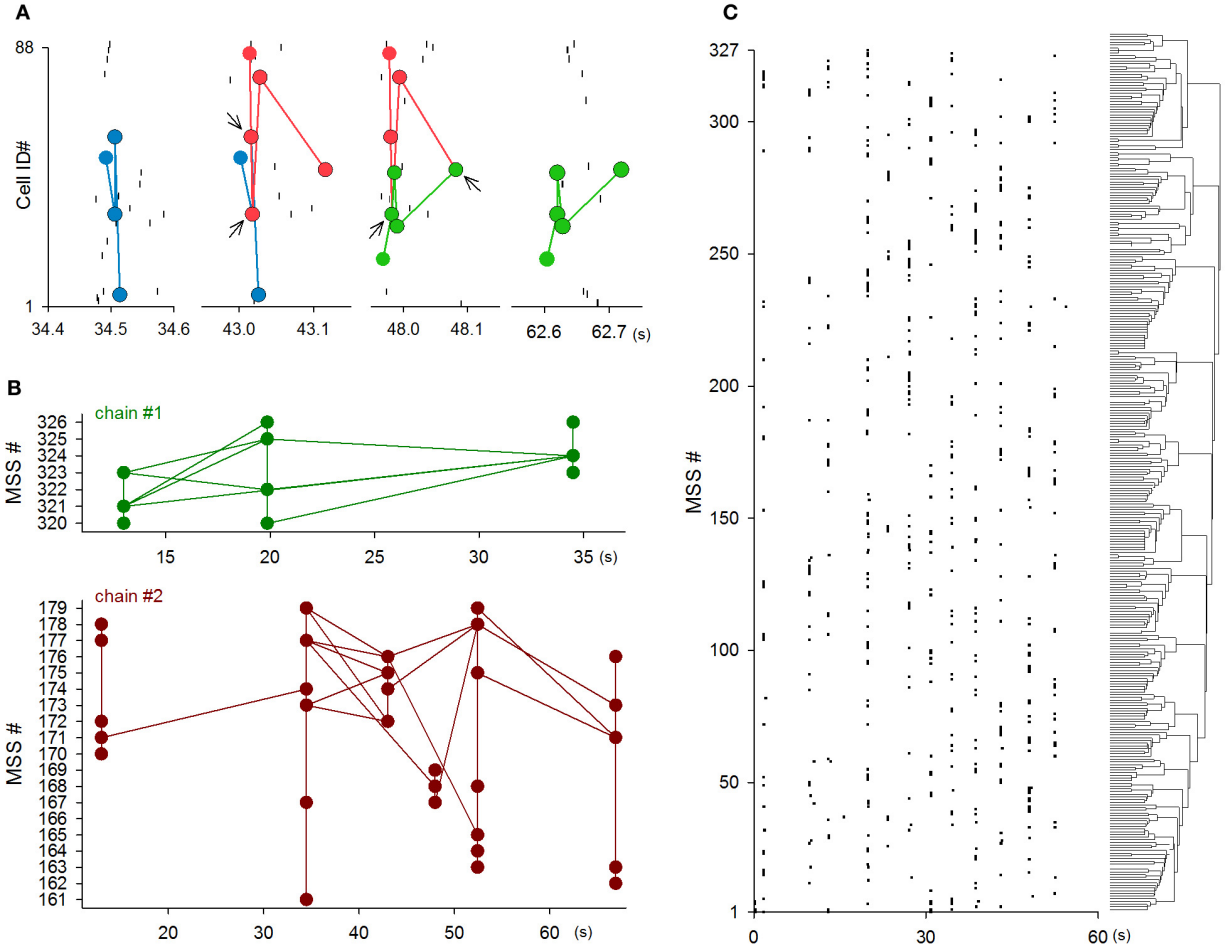
**Flexibility and complexity of MSSs. (A)** An example of an MSS chain. Spikes that consisted of MSSs were partially shared (i.e., relayed) by other MSSs. Arrows indicate the shared spikes, referred to here as core patterns. **(B)** Two examples of the time evolution of the chains that originated from single MSSs. **(C)** A rastergram of the onset of individual MSSs, the order of which were arranged along the dendrogram clustered by Ward's method.

The significant joint participation in MSSs, which was described above, is of importance in understanding MSS dynamics. Given that single neurons were recruited to different MSSs, the joint participation suggests the existence of “core” neuron groups that were shared with different MSSs. Indeed, we often encountered MSS series in which parts of MSSs were replayed by parts of other MSSs (Figure 12A).

We thus defined a MSS chain as a sequence of MSSs that shared at least two spikes. Under this definition, a transition from one MSS to another MSS through the shared spikes was referred to herein as ‘relay’. We also defined a core pattern as shared spikes at each relay step of a MSS chain. Figure 12A shows an example MSS chain in which three MSSs were relayed (from blue to red and from red to green) through two core patterns (indicated by arrows). Of 27,804 MSSs in 9 videos, 22,234 MSSs (80.0%) contained at least one core pattern. Single MSSs contained 1.44 ± 0.85 core patterns, and single core patterns were shared by 4.0 ± 4.2 MSSs (*n* = 3264 core patterns). The mean firing rate of neurons that were involved core patterns was 0.38 ± 0.26 Hz (*n* = 401), which was significantly higher than that of non-core neurons [0.08 ± 0.10 Hz, *n* = 427, *P* = 4.6 × 10^−8^, *t*_(826)_ = 5.57]. Figure 12B shows the whole dynamics of two representative MSS chains; each circle indicates a single MSS, and each line indicates a relay between two MSSs. Single videos included 26.1 ± 11.6 independent MSS chains (*n* = 9 videos). Single MSS chains consisted of 86.5 ± 284.3 MSSs, contained 53.6 ± 158.1 relay steps, and were 7.9 ± 4.4 s in length (*n* = 234 chains).

Relay steps did not always reflect simple relays between MSSs. The steps often exhibited divergent relays, in which core patterns in one MSS were subsequently used in two or more MSSs, or convergent relays, in which core patterns in two or more MSSs were simultaneously used in a subsequent MSS (see Figure 12B). Single chains contained 17.8 ± 56.6 divergent relays and 19.8 ± 61.7 convergent relays (*n* = 234 chains), suggesting that MSS chains constitute MSS subgroups. Therefore, based on core patterns between MSSs, we analyzed MSSs using Ward's method. Figure 12C depicts a dendrogram of MSSs in a representative video, indicating that MSSs were clustered into subgroups. Similar MSS cliqueness was observed in the other videos. As a whole, therefore, MSSs are not mere repetitions of precise firing patterns within specific sets of neurons, but they are parts of larger complex and flexible network dynamics.

## Discussion

In this work, we used fMCI with 2 ms temporal resolution and monitored CA3 network activity in cultured hippocampal slices. We searched MSSs using a template-matching method and analyzed them by comparing them to surrogates. We generated the surrogates using a spike-exchanging shuffling procedure, a randomization method that is believed to most reliably minimize false-positive errors because it does not collapse the firing rates of individual neurons or population modulation. We demonstrated that the temporal precision of MSSs in the hippocampus is high at the millisecond level. MSSs consisted of heterogeneous neurons or neuronal subsets. Moreover, we found core patterns across multiple MSSs, and the core patterns served as hubs through which MSSs are replaced with other MSSs and are sometimes split or joined together with other MSSs.

MSS-like fixed firing patterns have been reported in the neocortex of monkeys and rats (Abeles and Gerstein, 1988; Aertsen et al., 1991; Abeles et al., 1993; Prut et al., 1998; Mao et al., 2001; Cossart et al., 2003; Shmiel et al., 2006; Luczak et al., 2007). They have been thought to be experimental evidence supporting the synfire chain hypothesis, a theoretical framework for efficient spike propagation; however, the statistical significance of MSSs has been questioned by several studies (Oram et al., 1999, 2001; Baker and Lemon, 2000; Mokeichev et al., 2007). In this work, we simultaneously monitored spikes of approximately 100 neurons and confirmed that MSSs occurred in spontaneous activity more than chance. Importantly, the existence of MSSs was most significant at a spike jitter of zero frames. Thus, MSSs were repeated with spike precision of less than 2 ms. However, it is statistically difficult to find the true null hypothesis of temporal structures of spikes (Luczak et al., 2007). Therefore, we also tried to demonstrate the existence of MSSs by examining the properties of MSS-participating neurons. If MSSs are a stochastic product, neurons or neuron pairs with higher firing rates would be expected to participate more frequently in MSSs. Conversely, we found that, in the ratio scale, neurons with lower firing rates contributed more to MSSs. This result implies that MSSs are generated by network dynamics independent of the firing rates of individual neurons. Moreover, the number of neurons involved in single MSSs and the MSS length peaked at 6 neurons and 60 ms. These data also suggest that MSSs reflect organized network dynamics.

The majority of MSSs were generated by specific sets of neurons. Similar heterogeneous participations have been suggested by multi-electrode array recordings from cultures of dissociated mouse neocortical neurons (Sun et al., 2010). In this work, we further identified the core patterns that mediated MSS relays. The core patterns seemed to serve as hubs that generated a wide variety of MSSs that were dynamically associated with each other. The clustering of MSSs in the dendrogram suggests that single cells participate in different MSSs and that single MSSs are also involved in larger-scale MSS chains. This “MSS-family” concept is consistent with cell assembly dynamics and also with phase sequences of synfire chains, both of which occur together with network synchronization. However, the complexity of MSSs must be interpreted with caution. For instance, the mean number of neurons involved in single MSSs was 3.4. The mean number of total neurons monitored simultaneously was 91.7, whereas approximately 5000 CA3 neurons exist in a hippocampal slice culture (Kimura et al., 2011). This result means that we missed the vast majority of MSSs. Therefore, we underestimate both the true MSS size and the true complexity of MSS dynamics.

Although MSSs are theoretically accepted to be important for stable spike propagation through neuronal microcircuits that consist of weak and stochastic synapses (Abeles, 1991; Diesmann et al., 1999; Reyes, 2003), the physiological roles of MSSs in brain function remain unclear. Our work did not address this fundamental question, but it is intriguing to find that MSSs occurred during SW-Rs. We believe that MSSs underlie memory replay during SW-Rs. Manipulations of MSSs, i.e., artificial controls to increase or decrease MSSs, will help our understanding of the behavioral function of MSSs.

### Conflict of interest statement

The authors declare that the research was conducted in the absence of any commercial or financial relationships that could be construed as a potential conflict of interest.
